# Electrochemical Synthesis of Magnesium Hexaboride by Molten Salt Technique

**DOI:** 10.1155/2014/123194

**Published:** 2014-08-31

**Authors:** S. Angappan, N. Kalaiselvi, R. Sudha, A. Visuvasam

**Affiliations:** ^1^CSIR-Central Electrochemical Research Institute, Karaikudi 630006, India; ^2^Department of Physics, Selvam Arts and Science College, Namakkal 637003, India

## Abstract

The present work reports electrochemical synthesis of MgB_6_ from molten salts using the precursor consists of LiF–B_2_O_3_–MgCl_2_. An attempt has been made to synthesize metastable phase MgB_6_ crystal by electrolysis method. DTA/TGA studies were made to determine the eutectic point of the melt and it was found to be around 900°C. The electrolysis was performed at 900°C under argon atmosphere, at current density of 1.5 A/cm^2^. The electrodeposited crystals were examined using XRD, SEM, and XPS. From the above studies, the electrochemical synthesis method for hypothetical MgB_6_ from chloro-oxy-fluoride molten salt system is provided. Mechanism for the formation of magnesium hexaboride is discussed.

## 1. Introduction

Rare earth and alkaline earth metal borides belong to the group of nonoxide type metal-like compounds and have high melting point, high chemical stability, stable specific resistance, low expansion coefficient at certain temperature ranges, diverse magnetic orders, and high neutron absorbability [1, 2]. They have possessed excellent corrosion and wear resistance, chemical inertness, and thermal shock resistance more than that of oxide ceramics [3, 4]. The alkaline earth hexaborides were long thought to be simple polar semiconductors with single particle gap energy of several tenths of an eV and the energy gap is narrow as well as indirect band gap (Δ*E*_*g*_ = 0.0150 Ry).

Electrochemical synthesis of Mg–B system from molten salts is an economic feasible and environmental friendly way for the preparation of different binary phases [5]. Particularly, the Mg–B system was reported early [6, 7] to contain five phases, whereas Serebryakova [8] reported only four phases. Borides can exist as a wide range of compositions and display structural features, which depends strongly on the metal and boron ratio. Markowsky et al. proposed formation of three phases with higher B content as the result of thermal decomposition of MgB_2_: (1) MgB_6_, (2) unknown, and (3) MgB_12_ [9]. However, Duhart reexamined these data and claimed that phase 1 corresponds to MgB_4_ and phase 2 to MgB_6_ and the formation of MgB_12_ (phase 3) was not confirmed [10]. MgB_6_ and MgB_4_ do not exist as individual phases and obviously are metastable with rather long equilibration times. According to Somsonov et al. [11], Mg–B system has four stable borides: MgB_2_, MgB_4_, MgB_6_, and MgB_12_. Mg–B system contains the phases of MgB_2_, MgB_4_, MgB_6_, MgB_12_, and Mg_2_B_14_. So the Mg–B system is known as multiphase system. The aim of the present work is to study whether the thermodynamically unstable MgB_6_ [12, 13] could be prepared as thermally stable compound by electrochemical synthesis method.

## 2. Experimental Procedure

The mixture of the salts LiF (12.95 mol%), B_2_O_3_ (22.27 mol%), and MgCl_2_ (17.14 mol%) (analytical grade from Merck, India) was taken as an electrolyte in high-density graphite crucible and acts as an electrolyte cell as well as anode for the electrolytic process. The Molybdenum rod of 1 cm diameter fitted to a stainless steel rod is used as cathode. The crucible was filled with the stoichiometric quantities of electrolyte salts, which were dried at 500°C under argon atmosphere. The whole assembly was placed in an inconel reactor, which was kept in an electrical heating furnace with thermocouple. The experimental setup for the electrosynthesis of magnesium hexaboride is described elsewhere [14–17]. Then the salts were melted slowly under a continuous flow of argon gas. The melt was equilibrated at 900°C for one hour before proceeding electrolysis [15–17]. The bath was preelectrolyzed at 2.0 V for one hour to remove the impurities and moisture prior to electrolysis. The cathode was centrally positioned at the electrolytic cell. Experiment was carried out at current density of 1.5 A/cm^2^ with the molar ratio of Mg : B as 1 : 6. After 5 hours of electrolysis the cathode was removed and the deposit was cooled in atmosphere. The deposit was then scraped off and the electrolyte adhering to it was leached with warm 5% HCl solution. Finally washing was done with distilled water for several times, the weight of the deposit was determined, and the nature of the powder was analyzed.

The phase formation and the structural details of the synthesized compound were characterized by X-ray powder diffraction (XRD) using CuKα (*λ* = 1.541 Å) radiation with 2*θ* value range of 20 to 90 using PAnalytical X'pert powder diffractometer. Differential thermal analysis and thermogravimetric analysis (TGA/DTA) of the reaction mixture was done using Rigaku Thermal—Plus TG 8120 with heating speed 20°C/min in a flow of air. The Fourier transform infrared (FTIR) spectra were recorded in the range of 400 to 4000 cm^−1^ using Perkin Elmer UK Paragon—500 spectrometer. Scanning electron microscopy (SEM) was employed for the morphological studies using JEOL JSM 3.5 CF Japan make model. UV Visible Spectrophotometer was employed for the absorbance study using JASCO Model 7800 UV Visible Spectrophotometer. Studying the binding energy of boron and magnesium was done using X-ray photoelectron spectroscopy Thermo Scientific UK Multilab 2000.

## 3. Results and Discussion

**Figure 1:**
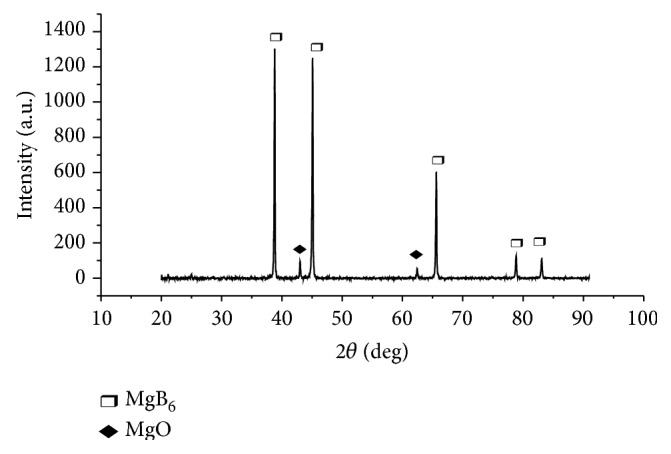
XRD pattern for MgB_6_.

Figure 1 presents the powder XRD pattern of the MgB_6_ synthesized by molten salt technique. The lattice constant value *a* = 4.114 Å is determined from the XRD data and is well matched with the reported value (*a* = 4.115 Å) [18–20] for MgB_6_ (JCPDS data card number 08-0421) existing in body centered cubic crystal structure (the space group Pm3m) [20]. But indexing the plans for MgB_6_ is difficult because the information on its lattice parameters and structure system is not available in 08-0421. The main building blocks of the MgB_6_ structure are B_6_ octahedra. Other than MgB_6_ some additional traces of MgO are also present at 2*θ* = 43 and 62.5° due to the partial oxidation of Mg [21]. The crystalline size is found to be 42 nm calculated by using Debye-Scherrer equation as follows:

(1) $$\begin{array}{c} D=\frac{k\lambda }{\beta \cos\theta }, \end{array}$$

where *k* is the Scherrer constant usually taken as 0.9, *λ* is the wavelength characteristics of the Cu-Kα radiation (*λ* = 1.5406 Å), *β* is the full width at half-maximum (FWHM) in radiations, *θ* is the reflecting angle, and *D* is the crystal size.

**Figure 2:**
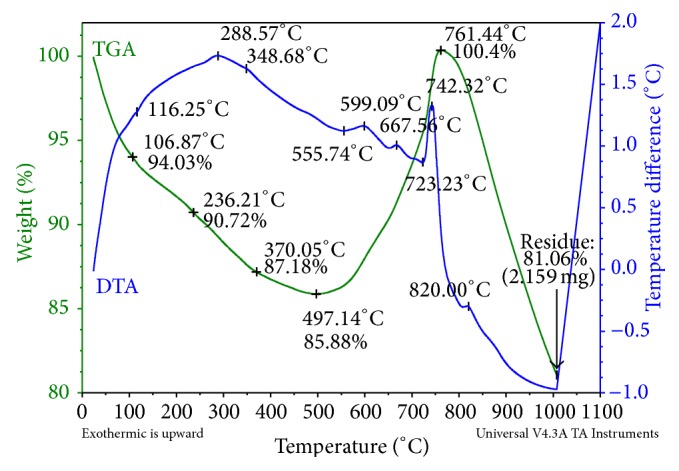
TG/DTA curve for MgB_6_.

**Figure 3:**
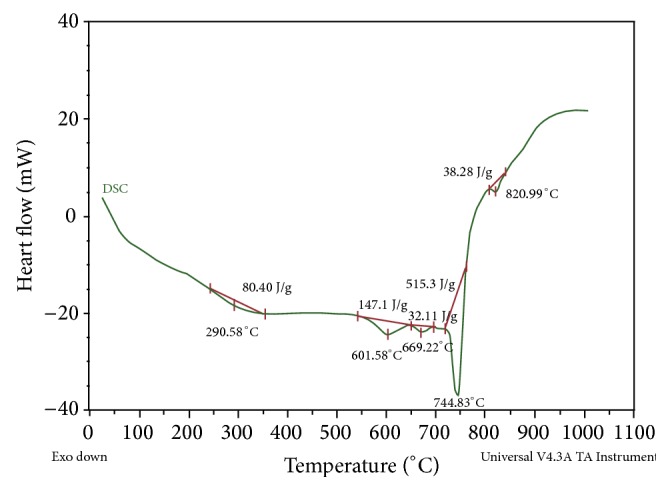
DSC curve for MgB_6_.

The TGA/DTA curve for the reaction mixture is shown in Figure 2. The figure showed that the eutectic point of the melt is found to be 820°C. The melt temperature is kept approximately 80°C higher than the eutectic point to reduce the melt viscosity. The LiF is used to increase the fluidity and electrical conductivity of the melt. Its decomposition potential is more cathodic than any other salts chosen. A gradual weight loss observed up to 497°C may be due to the removal of moisture and inbound water associated with the salts. The weight gain which is observed up to 761°C from 497°C, due to B_2_O_3_, begins to turn into liquid (melting point 450°C) in the heating process. These reactants whether in the liquid or gaseous state play a crucial role in determining the shape of the final product. Further, this weight gain is mainly due to the oxidation of the reactants (2) [22, 23]. MgCl_2_ stretches excess Mg and also increases the electrical conductivity of the melt. This excess Mg combines with O forming MgO (from residual B_2_O_3_). The formation of 2MgCl_2_·3B_2_O_3_ is due to the solid-state reaction between residual B_2_O_3_ and MgCl_2,_ turn into molten state (melting point of MgCl_2_: 708°C) is confirmed by an exothermic peak at 761°C. Finally, the reactants can be oxidized thoroughly at 761°C [24]. Further weight loss observed up to 1000°C is responsible for the transformation of the reactants into desired product. In the DTA curve, a sharp exothermic spike is observed at 742°C which may also be verified by confirming this process. This is gratuitous to the DSC curve (Figure 3) at 745°C; a sharp exothermic spine is observed that the heat of decomposition of salts is about 513.3 J/g.

The reaction was carried out for MgB_6_ in MgCl_2_–B_2_O_3_–LiF system; the main chemical reactions are

(2) $$\begin{array}{c} 2\text{Mg}{\text{Cl}}_{2}+3{\text{B}}_{2}{\text{O}}_{3}⟶2\text{Mg}{\text{Cl}}_{2}\cdot 3{\text{B}}_{2}{\text{O}}_{3} \end{array}$$

(3) $$\begin{array}{c} 2\text{Mg}{\text{Cl}}_{2}\cdot 3{\text{B}}_{2}{\text{O}}_{3}⟶\text{Mg}{\text{B}}_{6}+\text{MgO}+4{\text{O}}_{2}\left(\text{g}\right)+2{\text{Cl}}_{2}\left(\text{g}\right)↑ \end{array}$$

The overall reaction is

(4) $$\begin{array}{c} 2\text{Mg}{\text{Cl}}_{2}+3{\text{B}}_{2}{\text{O}}_{3}⟶\text{Mg}{\text{B}}_{6}+\text{MgO}+4{\text{O}}_{2}+2{\text{Cl}}_{2}\left(\text{g}\right)↑ \end{array}$$

Trace amount of the unreacted intermediate MgO was present in the synthesized compound as 0.6% and the remaining 99.4% was MgB_6_ as depicted from XRD pattern.

**Figure 4:**
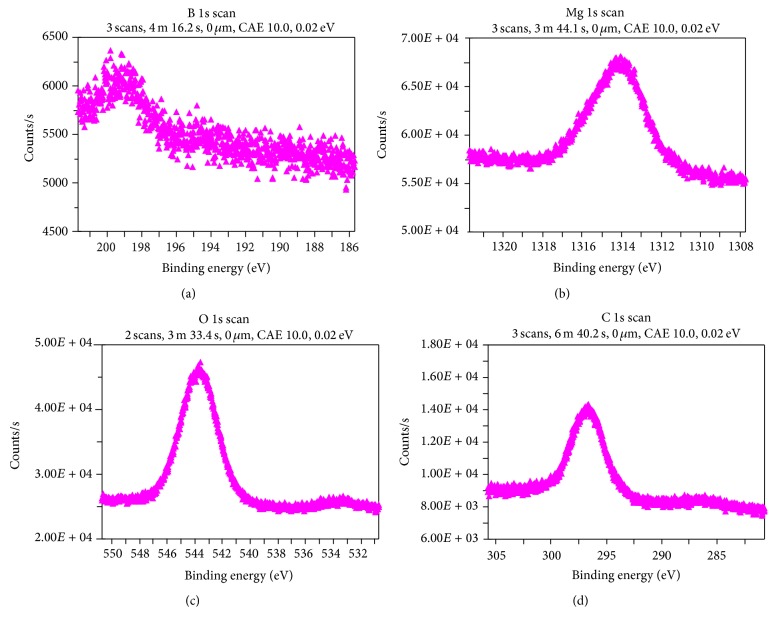
XPS spectra for (a) B 1s, (b) Mg 1s, (c) O 1s, and (d) C 1s.

The XPS spectrum for B 1s is shown in Figure 4(a). The higher binding energy value for B 1s exists at 198.6 eV. This reflects contributions from both trigonal BO_3_ and tetrahedral BO_4_ groups. The electron transfer would come from trigonal B 1s to B–O *σ* ^*^ orbital and from the unfilled tetrahedral B 2p orbital to B–O *σ* ^*^ [25, 26]. This B 1s → *σ* ^*^ resonance as expected for sp^2^-bonded boron incorporated in the crystal [27].  Figure 4(b) shows the Mg 1s spectra for MgB_6_ at 1314 eV revealed that the auger spectral distribution over an extruded energy range far from the threshold; there is an extra energy for Mg rich compound [28].  Figure 4(c) shows the O 1s spectrum for MgB_6_ existing at 544 eV. This may be due to the core-hole Rydberg states containing O 1s → *σ* ^*^ resonance [29, 30].  Figure 4(d) shows C 1s spectrum at 296 eV and reveals energy transitions between a carbon core level and an antibonding *π* ^*^ molecular orbital [31]. The surface is contaminated due to exposure to air during the processing of the sample.

The Mg^2+^ cations in MgB_6_ complex species have C_6*v*_ pyramidal structures interacting with a planar hexagonal B_6_^2−^ dianion. The bonding between these two may be due the electrostatic attraction [12].

**Figure 5:**
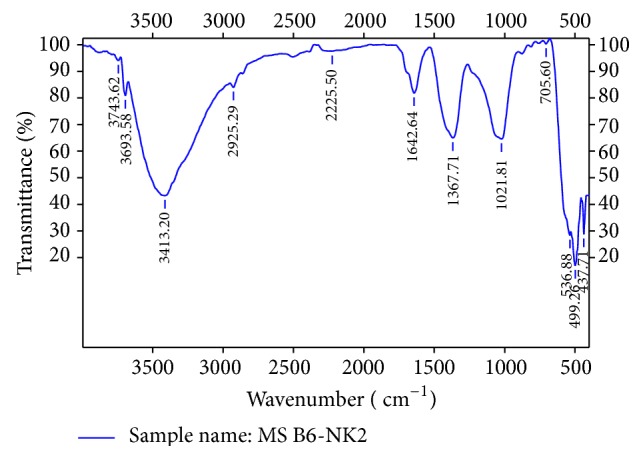
FTIR spectrum of MgB_6_.

The FTIR spectrum of MgB_6_ is shown in Figure 5. The O–H stretching vibrations of water crystallization are represent at 3743 and 3413 cm^−1^, respectively. The absorption at 2225 cm^−1^ is assigned to O–H stretching vibration of cluster of water molecules of crystallization, respectively. The characteristic peak of Mg–B is observed at 1642 cm^−1^ [32]. Longitudinal optic mode frequency of Mg–O is observed at 705 cm^−1^; this MgO as impurity phases is also observed in the XRD pattern. The bending vibration for MgB_6_ is observed at 437 cm^−1^. The bending mode of Mg–B of BO_4_ anion is assigned to 499 cm^−1^. The asymmetric stretching vibration of Mg–B of BO_4_ anion is observed at 1021 cm^−1^. The asymmetric stretching vibration of B–O bond of trigonal BO_3_ units is observed at 1367 cm^−1^. The frequencies observed in the spectrum are in good agreement with the reported values [33, 34].

**Figure 6:**
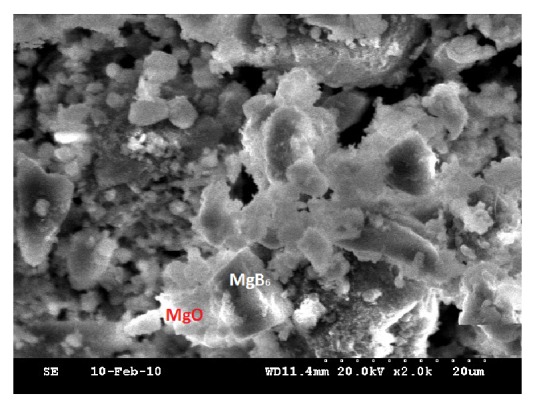
SEM image of MgB_6_.

The SEM image of the sample is shown in Figure 6. The microstructure indicates that the particle diameters are in the range 4–8 *μ*m. From the microstructure, the molten regions are clearly visible, which give the clear indication of MgB_6_ formation. The white regions in SEM represent the impurity phase MgO present in the sample [35, 36]. The present study reveals the formation of MgB_6_ phase as cubic crystal structure.

The mechanism of hexaboride formation was proposed by many authors [12, 14–17, 37–47]. According to Li and Jin, the negative charged boron atoms and the positive charged alkaline earth metal atoms form complexes of M^2+^ metal cation and B_6_^2−^ dianion due to electrostatic attraction. They also suggested that the metal cations M^2+^ have definite role on stabilizing the B_6_^2−^ dianion [12]. Kaptay and Kuznetsov reported that the boron components are dissolved in ionic form in the melt, to form boride phase on the cathode joint with metal cations [37]. Jose et al. reported the “unstable stoichiometric way” for the deposition of Barium hexaboride [14]. We reported earlier that the electrolytically dissociated metal and B ions deposit on the cathode as CeB_6_ and SmB_6_, respectively [15, 16]. We also reported in our earlier study on CaB_6_ that the calcium and boron are reduced at the cathode to form submicron sized crystals [17]. As reported by Chen et al. [38], the formation of MgB_6_ at 900°C as one of the secondary phases along with MgB_4_ due the volatile nature of Mg at this temperature resulted in Mg deficiency on in situ Cu doping of MgB_2_. The commonly accepted mechanism of boron deposition in molten salts is a single-step three-electron electrochemical reaction [39–43]. Gloor et al. investigated the multiexciton bound state of molecules in divalent hexaborides. They proposed that the larger energy gain per one electron-hole pair decreases the semiconducting gap and produced intermediate phase. This may be the reason for the formation of intermediate phase MgB_6_ [44]. Li et al. described the diffusion of Mg vapour into boron creating a complex of Mg–B supersaturated solution, encompassing the formation of nonequilibrium MgB_6_ [45]. Lee et al. and J. Q. Li also authenticated with S. Li [45] and postulated that the path of the reaction of supersaturated Mg–B cluster complex via spinodal decomposition leads to the formation of hypothetical phase MgB_6_ [46, 47].

## 4. Conclusion

In summary, the electrochemical synthesis of hypothetical magnesium hexaboride by molten salt technology is presented. Various mechanisms for the formation of magnesium hexaboride are discussed. It is believed that the supersaturated MgB_6_ cluster complex is postulated for the metastable magnesium hexaboride compound. Further experimental evidence is more needed to explore the thermodynamically unstable magnesium hexaboride.
